# Fecal Short Chain Fatty Acids and Dietary Intake in Italian Women With Restrictive Anorexia Nervosa: A Pilot Study

**DOI:** 10.3389/fnut.2018.00119

**Published:** 2018-11-29

**Authors:** Enza Speranza, Iolanda Cioffi, Lidia Santarpia, Concetta Del Piano, Carmela De Caprio, Marianna Naccarato, Maurizio Marra, Emilia De Filippo, Franco Contaldo, Fabrizio Pasanisi

**Affiliations:** ^1^Department of Clinical Medicine and Surgery, Federico II University Hospital, Naples, Italy; ^2^Interuniversity Centre for Obesity and Eating Disorders (CISRO), Federico II University Hospital, Naples, Italy

**Keywords:** anorexia nervosa, SCFA, malnutrition, energy expenditure, diet

## Abstract

Nutritional disorders such as Anorexia Nervosa (AN) can shape the composition of gut microbiota and its metabolites such as short chain fatty acid (SCFA). This study aims to compare fecal SCFA along with dietary intake of women with restrictive AN (r-AN = 10) and those of sex-matched lean controls (C = 8). The main fecal short chain fatty acids (SCFA) were assessed by gas chromatography equipped with a flame ionization detector. All participants completed 7-day food record and underwent indirect calorimetry for measuring resting energy expenditure (REE). Butyrate and propionate fecal concentrations were significantly reduced in r-AN patients compared to controls. The intake of carbohydrate and fat was significantly lower in r-AN patients than controls as well as energy intake and REE; whereas the amount of protein and fiber did not differ between groups. These preliminary results showed that r-AN patients had a reduced excretion of fecal SCFA, likely as a mechanism to compensate for the lower energy and carbohydrate intake observed between groups. Therefore, further studies need to be performed in patients with AN to explore the link between nutritional disorders, gut microbiota and its metabolites.

## Introduction

Nutritional disorders such as anorexia nervosa (AN) can shape the composition and activity of the gut microbiota (1). Restrictive Anorexia nervosa (r-AN) is the most serious clinical subtype of AN characterized by severe dietary restriction and/or other weight loss behaviors (2), but the pathophysiological mechanisms are still unclear. Generally, patients with r-AN have insufficient energy intake with inadequate intake of certain macronutrients and micronutrients (3); however, malnutrition secondary to eating disorders develops slowly over time due to adaptive metabolic mechanisms to chronic underfeeding (4). A growing body of evidence recognizes the role of gut metabolites in affecting host metabolism and appetite through a variety of pathways (5), many of which are dependent on the diet of host, such as short chain fatty acids (SCFA) (6). SCFA are mainly produced by the fermentation of indigestible carbohydrates, especially dietary fibers and resistant starch, in the large intestine and are an important source of energy for colonocytes (7). The most abundant are acetic, propionic and butyric acids, representing 90–95% of the total SCFA (8). However, the final balance of SCFA production can be affected by some mechanisms such as the bacterial cross-feeding (9) beside substrate cross-feeding. Previous studies conducted in subjects with r-AN reported specific alteration of the gut microbiota and its metabolites when compared to both obese and lean subjects (10); specifically, SCFA increased in overweight/obese individuals (11) and decreased in AN subjects (12–14) than lean subjects. Several mechanisms as colonic SCFA absorption, colonic transit time, and differences in dietary intake and/or in colonic microbiota can modulate fecal SCFA concentration. Hence, the aim of this pilot study was to assess fecal SCFA concentration along with dietary intake, collected by 7-day food records, in women with r-AN compared to lean subjects.

## Material and methods

### Participants recruitment

The present study was part of an observational study that explored gut microbiota and its metabolites in different diseases condition (15). In this pilot study, we compared fecal SCFA and dietary intake provided by r-AN patients to a control group, asking them to collect their feces after recording food for consecutive 7 days. Fourteen young women with diagnosis of r-AN, according to Diagnostic and Statistical Manual of Mental Disorder (DSM)—V, were screened for the recruitment in this study from outpatient visit at the Internal Medicine and Clinical Nutrition Unit of Federico II University Hospital in Naples, Italy. At the enrollment 4 participants dropped out due to personal reason, therefore 10 r-AN women were finally recruited. On the other hand, 10 healthy sex- and age -matched healthy subjects were screened for the control group (C), but only 8 agreed to participate in this study. Participants with history of digestive disease such as inflammatory bowel disease, use of antibiotics or probiotics within 3 months of study participation, habits of smoking, intensive physical activity and use of laxatives during the week before were excluded. The protocol was approved by the Local Ethical Committee of the Federico II University Hospital (Prot. Numb.155/14). All subjects gave written informed consent in accordance with the Declaration of Helsinki.

### Energy intake and nutrients assessment

Participants were instructed by a registered dietitian to fill in a food diary for 7 consecutive days and were trained for estimation of food portions by using household measurement tools. Specifically, participants were taught how to use tools such as bowls, cups, spoons, and plates to quantify food portions. In addition, pictures of varying portion sizes (small, medium, and large) of most widely consumed foods were shown to participants. A dedicated dietitian reviewed the completed 7-day food diary upon return for clarification of portions, missing or unclear data, and food preparation methods. All diaries were calculated using the WINFOOD database (3.4 version; Medimatica, Teramo, Italy).

### Resting energy expenditure (REE) measurement

REE was measured by indirect calorimetry (16) (Vmax29, Sensor Medics, Anaheim, California) with a ventilated hood and canopy system. Measurements of REE were made early in the morning and patients were instructed to follow a standardized fasting procedure on the day before (i.e., abstention from alcohol and intense physical activity). The indirect calorimetry was checked by burning ethanol, then oxygen and carbon dioxide analyzers were calibrated using nitrogen and standardized gases (mixtures of nitrogen, carbon dioxide and oxygen) before every run (17). Patients were asked to lie down for at least 10 min in a quiet environment and at room temperature of 23–25°C; then oxygen and carbon dioxide production were determined for 30 min. EE was then calculated using the abbreviated Weir's formula, neglecting protein oxidation (18).

### Short chain fatty acid (SCFA) measurement

Fecal samples were collected from all participants, stored in sterile plastic hermetically sealed boxes and processed for the analysis. One gram of feces was suspended in 5 ml of distilled water and mixed per 5 min. The fecal sample was homogenized in perchloric acid (0.15 mol/L), and centrifugated at 4,000 rpm for 10 min. The aqueous fecal phase (980 μl) was collected and 20 μl of methacrylic acid (2.5 mMol/ ml) was added. The concentrations of SCFA (butyrate, acetate, and propionate) were measured using a gas chromatography equipped with a flame ionization detector (Hewlett Packard 5890 Series II) (19), by injecting 1 μl of stool sample into the capillary column (Supelco SPBTM 30 m × 0.25 mm × 0.25 mm) and data were evaluated using an integrator manual (Hewlett Packard 3396 Series II).

### Statistical analysis

All data are shown as mean ± standard deviation (SD), otherwise stated. All dependent variables were controlled for normal distribution by Shapiro-Wilk test. If the distribution of a variable was skewed, it was log-transformed prior to analyses and back-transformed before presentation. Differences in SCFA were analyzed by using Wilcoxon's rank test. Pearson's correlations were used to test associations between variables with normal distributions; otherwise, Spearman's correlation was applied. Statistical analyses were performed using SPSS version 18.0 (Chicago, IL) and significance level was set at the *p* < 0.05.

## Results

All participants recruited completed the study, however 2 out 8 control subjects did not deliver food diary. Both groups differed for body weight (r-AN = 37.3 ± 3.8 vs. C = 55.8 ± 3.4 kg; *p* = 0.01), BMI (r-AN = 14.5 ± 1 vs. C = 22.1 ± 1.9 kg/m2; *p* = 0.01) and, even though the range of age was similar between groups (20–32 years), age resulted different (r-AN = 23.5 ± 2.8 vs. C = 29.2 ± 2.9 years; *p* = 0.02). As expected, the total energy intake was significantly lower in r-AN patients than C and macronutrient composition differed significantly between groups as reported in Table 1. r-AN had a lower amount of carbohydrate and fat compared to controls, while the intake of both protein and dietary fiber was similar. Likewise, trace elements and vitamins intake did not significantly differ between groups. None of the participants reported any alcohol consumption. As expected, REE measured by indirect calorimetry was reduced for r-AN than C; but those values were compliant with their self-reported energy intake.

**Table 1:** Dietary intake and measured REE in r-AN patients and controls.

**r-AN**	**Range (min-max)**	**Controls**	**Range (min-max)**	***p*-value**
	**Mean, SD**		**Mean, SD**		
Energy intake (kcal/d)	1032 ± 330	632–1479	1597 ± 344	1197–2238	0.02
Protein (g)	61.9 ± 19.4	26–95	78.6 ± 22.4	56–112	Ns
Fat (g)	28.7 ± 10.5	15–51	48.1 ± 19.9	19–76	0.03
SFA (g)	7.1 ± 5.2	2–18	13.6 ± 8.0	3–26	Ns
MUFA (g)	11.8 ± 4.6	4–20	22.0 ± 10.5	5–34	0.01
PUFA (g)	2.7 ± 0.9	1–4	5.7 ± 2.4	3–9	0.03
Carbohydrate (g)	140.3 ± 76.1	43–284	220.7 ± 44.4	175–296	0.02
Starch (g)	78.6 ± 46.7	0.30–141	125.2 ± 44.4	48–171	Ns
Oligosaccharides (g)	48.1 ± 33.8	5–132	77.9 ± 41.3	14–125	Ns
Fiber/1,000 kcal (g)	14.3 ± 9.1	5–33	13.2 ± 4.6	6–19	Ns
Total fiber (g)	15.1 ± 11.4	5–39	21.0 ± 8.3	9–30	Ns
MREE (kcal/d)	872 ± 155	734–1165	1393 ± 99	1245–1483	0.01
QR	0.90 ± 0.06	0.8–1.1	0.86 ± 0.08	0.8–0.9	Ns

*Data were obtained from 7-day food record and are shown as mean ± SD; r-AN (n = 10); C (n = 8).*.

*MREE, Measured Resting Energy Expenditure; SFA, Satured Fatty Acid; MUFA, Monounsaturated Fatty Acid; PUFA, Polyunsaturated Fatty Acid; Ns, not significant*.

**Figure 1:**
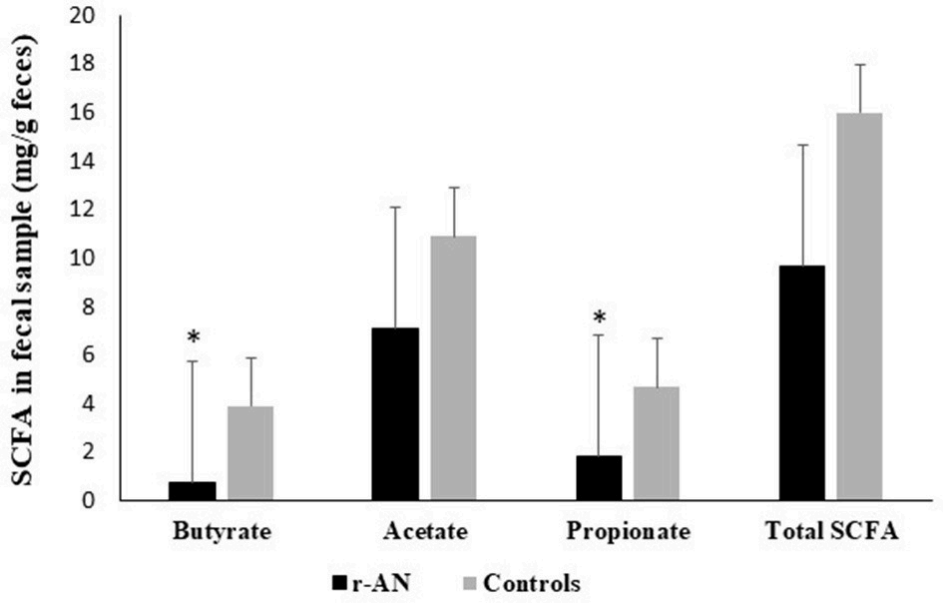
Fecal SCFA concentrations in subjects with anorexia nervosa and healthy controls. ^*^p < 0.05.

The concentrations of fecal SCFA were reported in Figure 1, showing that butyrate and propionate were significantly lower in r-AN patients than controls; whereas, acetate and total SCFA concentration did not differ. Correlation tests were run for weight, dietary intake and SCFA analyzed in the study; observing that weight and BMI were positively correlated to both butyric (*r* = 0.76 and *r* = 0.72, *p* < 0.01) and propionic acid (*r* = 0.71 and *r* = 0.77; *p* < 0.01). As concern dietary intake, the amount of fat as well as of starch were directly correlated to propionic (*r* = 0.70 and *r* = 0.59; *p* < 0.01) and acetic acid (*r* = 0.70 and *r* = 0.61; *p* < 0.01); while butyrate was correlated to carbohydrates and oligosaccharides (*r* = 0.70 and *r* = 0.61; *p* < 0.01).

## Discussion

The aim of this pilot study was to assess fecal SCFA and self-reported dietary intake in a small group of r-AN patients compared to control subjects. Our preliminary findings showed that fecal butyrate and propionate concentrations as well as dietary intake differed between the two groups.

Anorexia is characterized by an altered gut microbiota composition and activities. In fact, so far, several studies have explored the fecal excretion of SCFA in r-AN patients, compared to healthy participants, showing reduced fecal concentrations of mainly propionic and butyric acid (12–14), according to our results. On the other hand, acetic acid was the most abundant and did not differ between groups. Interestingly, Borgo et al. (14) assessed SCFA concentrations in plasma as well, reporting that acetate was the only metabolite found; however, no significant relationship was observed between systemic and fecal concentrations.

Fecal SCFA concentration can be influenced by nutrients availability of the diet (20). Therefore, a hypocaloric diet typically characterized by high protein and low carbohydrate intake could result in lowering fecal SCFA levels in patients with r-AN (12–14); likely by developing improved mechanisms in absorption and digestion of nutrients in the gut (21) and/or prolonging the colonic transit time due to constipation (22). Data, obtained by 7-day food records, revealed that diet in r-AN patients was low in fat and carbohydrates, but not in protein and dietary fibers, in comparison to control subjects, as already reported (3, 13). Although dietary fibers are the main substrate for bacteria fermentation in the colon, it is likely that also other indigestible dietary substrates reached the colon in much smaller amount (10), due to the overall food reduction (13) that occurred in undernourished patients. Furthermore, it has been reported that a lower amount of carbohydrate, specifically starch, in the diet significantly decreased numbers of the butyrate-producing species, with a concomitant reduction in butyrate formation and excretion in the feces (23).

The present study has several limitations. First and most important, the sample size is small and may therefore affect our results; and another limitation is that both groups differ for the mean age, although they had the same age range (20–32 years). In conclusion, these preliminary results confirmed that women with r-AN show a reduced excretion of fecal butyrate and propionate, most likely to compensate for the lower energy and carbohydrate intake, as reported previously. However, these results need to be further investigated to clarify the link between nutritional disorders, gut microbiota and its metabolites.

## Author contributions

FP, FC, and ES designed the study. CDC, ES, and MN collected the data. MM and IC analyzed the data. EF, IC, and LS interpreted the data. IC and ES wrote the manuscript. All authors participated to the discussion of results and commented the manuscript and agreed to be accountable for all aspects of the work.

### Conflict of interest statement

The authors declare that the research was conducted in the absence of any commercial or financial relationships that could be construed as a potential conflict of interest.
